# Dealing with phosphorus deficiency: contrasting strategies in marine phytoplankton and bacteria

**DOI:** 10.1093/ismeco/ycag035

**Published:** 2026-02-20

**Authors:** Erick Delgadillo-Nuño, Eva Teira, Emilio Fernández, Maider Justel-Díez, Danilo Di Leo, Daniel Lundin, Jarone Pinhassi, Sandra Martínez-García

**Affiliations:** Departamento de Ecoloxía e Bioloxía Animal, Centro de Investigación Mariña da Universidade de Vigo (CIM-UVigo), 36310 Vigo, Spain; Departamento de Ecoloxía e Bioloxía Animal, Centro de Investigación Mariña da Universidade de Vigo (CIM-UVigo), 36310 Vigo, Spain; Departamento de Ecoloxía e Bioloxía Animal, Centro de Investigación Mariña da Universidade de Vigo (CIM-UVigo), 36310 Vigo, Spain; Departamento de Ecoloxía e Bioloxía Animal, Centro de Investigación Mariña da Universidade de Vigo (CIM-UVigo), 36310 Vigo, Spain; Centre for Ecology and Evolution in Microbial Model Systems, Linnaeus University, Kalmar, SE-39182, Sweden; Centre for Ecology and Evolution in Microbial Model Systems, Linnaeus University, Kalmar, SE-39182, Sweden; Centre for Ecology and Evolution in Microbial Model Systems, Linnaeus University, Kalmar, SE-39182, Sweden; Departamento de Ecoloxía e Bioloxía Animal, Centro de Investigación Mariña da Universidade de Vigo (CIM-UVigo), 36310 Vigo, Spain

**Keywords:** microbial communities, phytoplankton, bacteria, interactions, phosphorus, nitrogen, metatranscriptome, differential gene expression, coastal systems

## Abstract

Phosphorus (P) and nitrogen (N) are essential nutrients for microbial growth, playing crucial roles in regulating the biological productivity of marine ecosystems. Over the last decades, the relatively higher increase in anthropogenic N compared to P inputs is causing a continuous increase in the N:P supply ratio to the global biosphere. The high N:P ratio of riverine discharge may seasonally cause P limitation in estuaries and river-dominated continental shelf waters. We conducted a mesocosm experiment simulating a P-deplete and a P-replete riverine discharge to coastal waters in NW Spain to assess the functional response of marine microplankton using a metatranscriptomic approach. By examining the expression of 40 well-documented genes related to P-metabolism in prokaryotic and eukaryotic gene expression, we uncovered pronounced changes in microbial P-metabolism induced by riverine N:P ratio in this productive system. Remarkably, heterotrophic bacteria and eukaryotic phytoplankton exhibited contrasting phosphate metabolism strategies in response to P deficiency, with the former mostly expressing genes coding for high-affinity transporters and the latter mostly transcribing genes related with low-affinity transporters. Our results also highlight distinct regulatory and adaptive mechanisms across different members of the prokaryotic and eukaryotic communities when exposed to varying P concentrations. Our findings shed light on the broader ecological and functional roles of these genes in nutrient cycling within aquatic ecosystems, with potential application for the design of diagnostic tools for P status in coastal productive systems.

## Introduction

Microbial communities are important components of marine food webs and mediate major biogeochemical cycles [e.g. phosphorus (P) and nitrogen (N) cycles] in estuarine and coastal waters [1]. Microbial roles in biogeochemical processes are dependent on the activities and behaviors associated with their functional genes [2]. P and N are essential biogenic elements for microbes because of the pivotal role they hold in numerous cellular processes, including energy transfer, protein synthesis, and genetic coding [3, 4]. In fact, the availability of N and P shapes not only the taxonomic composition of the microbial communities in the ecosystem but also the nutrient acquisition and utilization strategies of the microbial communities present. Thus, the presence/absence and diversity of specific microbial functional genes have been suggested as useful indicators of nutrient load of a given ecosystem [5].

A global increase of molar N:P in anthropogenic nutrient inputs to the global biosphere has been registered in the last decades [6]. The generalized increase in the N:P ratio in rivers draining into the world’s oceans appears to be associated with the concurrent 2.5-fold increase of molar N:P in fertilizers and higher efficiency in P compared to N retention during processing and transport in soils, groundwater, streams, rivers, and reservoirs [7]. These altered nutrient inputs may promote seasonal P stress and affect microbial plankton dynamics in coastal systems dominated by N-enriched freshwater inputs [8–12]. Microbial responses to inorganic P (Pi) deficiency differ depending on the strategies utilized to cope with such limitations. Phytoplankton and heterotrophic bacteria are known to have distinct nutrient requirements and uptake efficiencies and heterotrophic bacteria have been described as better competitors than phytoplankton for P uptake [9, 13, 14], particularly at very low P concentrations [15] and, importantly, when they are not limited by organic C [8, 16–20]. Hence, phytoplankton and bacteria appear to have evolved different P-acquisition strategies and adaptations to cope with P deficiency that might range from P conservation to P niche-partitioning.

Depending on inorganic phosphate (Pi) availability, phytoplankton and bacteria may shift the strategy to incorporate Pi. On one hand, under non-limiting Pi concentrations, low-affinity–high-velocity transport systems (encoded by *pit* and *npt*, the Na-dependent phosphate transporter) are preferably utilized [21, 22]. On the other hand, under limiting P conditions (typically below 1 μM for eukaryotes [23, 24] and 0.1 μM for prokaryotes [25, 26]), the first response to stress in both phytoplankton and bacteria is the activation of the phosphate regulon (e.g. *prtA* and *PtPSR* genes in eukaryotes and *phoB/phoR/phoU* genes in prokaryotes) which modulates the P-starvation response and enables microbial communities to utilize high-affinity–low-velocity transport systems (encoded by *pstABC* and *pstS* genes) [21–27]. Willsky and Malamy [27] reported that, with respect to Pi uptake reaction rates, *K*_m_ and *V*_max_ values were, respectively, two orders of magnitude and three times lower for high- compared to low-affinity systems in *Escherichia coli*. Phytoplankton and bacteria have the capability to utilize both kinds of Pi transport systems, as extensively shown by available genomic datasets [21, 28–33], thus highlighting a constant trade-off between binding strength and speed under changing environmental conditions and nutrient demands. Thus, the use of high-affinity Pi transporters is a widespread strategy to take up Pi under limiting conditions in heterotrophic bacteria [34], cyanobacteria [35–38], and eukaryotic phytoplankton [3, 23, 39, 40].

On the other hand, both phytoplankton and bacteria may retrieve P from organic compounds such as phosphomonoesters (through alkaline phosphatases encoded by *phoX*/*phoA*/*phoD* genes) [21, 22, 37, 41–44] and phosphonate (hydrolysis and transport encoded by *phnXYWZ/phnA*, *phnGHIJKLM*, and *phnCDE* genes) [37, 45–48]. Similarly, under P-limiting conditions, eukaryotic and prokaryotic marine plankton have also been seen to mobilize and degrade internal polyphosphate (catalytic activity encoded by *ppk*, *ppX*, and *ppA* genes) [22, 49], to utilize alternative inorganic P forms like phosphite (*ptxABCD* genes), and to substitute P by sulfur in cell membrane phospholipids (phospholipase enzyme encoded by the *plcP* gene) [42, 50, 51]. Overall, both microbial compartments have been shown to obtain P utilizing different sources and mechanisms. However, little is known about the specific set of functional genes preferentially expressed by marine phytoplankton and bacteria in natural microbial communities growing under P-limiting conditions.

The aim of the present work is to investigate the potential mechanisms utilized by natural microbial communities to acquire phosphorus in a coastal system subjected to continental inputs with high N:P ratios. We used a mesocosm experiment approach to investigate the changes of P-related microbial functions after P-deplete and P-replete riverine inputs. Our comparative metatranscriptome analysis comprised a collection of 64 genes (Supplementary Table 1) previously used as informative markers for P acquisition and utilization by both phytoplankton and bacteria. Given that both microbial compartments are known to have distinct nutrient requirements and uptake efficiencies, we hypothesized that P limitation caused by P-deplete riverine inputs would promote contrasting P-acquisition strategies among different members of the phytoplankton and bacterial communities.

## Materials and methods

### Experimental design

**Figure 1:**
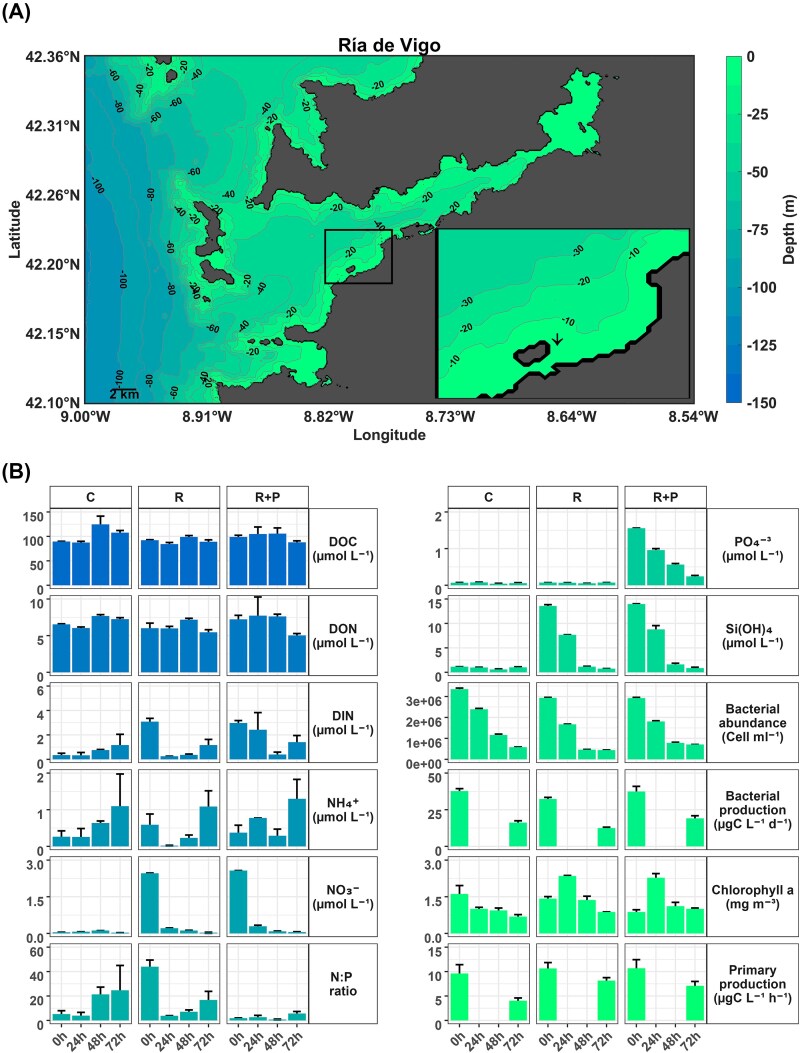
(A) Map showing the Ría de Vigo bathymetry (NW Iberian Peninsula) and the location of the mesocosm experiment conducted during spring 2019 at the Toralla Marine Science Station (ECIMAT). (B) Temporal changes in dissolved organic carbon (DOC), dissolved organic nitrogen (DON), dissolved inorganic nitrogen (DIN), ammonium (NH₄^+^), nitrate (NO₃^−^), phosphate (PO₄^3−^), silicate (Si(OH)₄^+^), N:P ratio, bacterial abundance, bacterial production, chlorophyll-*a* concentration, and primary production across the three mesocosm treatments: C (*Control*), R (*River*), and R + P (*River + P*). Data represent mean ± standard error (*n* = 3). Please note that PP and BP were not quantified at 24 and 48 h.

To evaluate the interactions shaping microbial community responses to riverine inputs, we performed a mesocosm experiment during spring 2019, using three 500-l UV-stabilized polyethylene bags maintained under *in situ* conditions using a floating structure anchored nearby the Toralla Marine Science Station (ECIMAT) in the Ría de Vigo (42° 12′ 7.081″ N, 8° 47′ 53.830″ W; Fig. 1A). See Supplementary Methods and Justel-Díez [52] for additional information. The mesocosm bags contained marine water with a 10% riverine addition (both pre-filtered through a 200-μm-pore-size mesh) for the *River* (R) and *River + P* (R + P) treatments. Thus, three treatments were established in the three mesocosm bags: *Control* (C) representing natural P-deplete conditions [dissolved inorganic nitrogen (DIN) = 0.3 μM, dissolved inorganic phosphorus (DIP) = 0.07 μM at the beginning of the experiment]; R that naturally contains N and thus represents P-deplete conditions (DIN = 3 μM, DIP = 0.07 μM at the beginning of the experiment); and R + P with an addition of phosphorus and thus representing P-replete conditions (DIN = 3 μM, DIP = 1.6 μM at the beginning of the experiment). Nutrient delivery by riverine inputs in this area has been reported to be ~1500 mg N m^−2^ year^−1^ and riverine waters have been registered to contain N:P ratios from 28 to >1000 [12, 53]. By contrast, N:P ratios of seawater in the area are typically <17 [54]. Our experimental design has the aim of simulating a riverine input and is therefore an important addition of N to coastal waters with (R treatment) and without P limitation (R + P treatment). Thus, the rationale for selecting an addition of 1 μM P for the R + P treatment was to avoid P limitation in this treatment. Due to logistic and operational limitations, each of the treatments was carried out in one 500-l bag, and we therefore acknowledge the derived statistical limitation. However, the extremely contrasting responses observed in the experimental treatments make us confident on the representativeness of the results.

### Inorganic and organic nutrient determinations

The concentration of dissolved organic carbon (DOC) and dissolved organic nitrogen (DON) was estimated using a Shimadzu TOC-V Total Organic C Analyzer with a Shimadzu TNM-1 Total N Unit. Nitrate (NO₃^−^), phosphate (PO₄^3−^), and silicate (SiO₂H₄) were calculated by the colorimetric method [55] and ammonium (NH₄^+^) by fluorometry [56] using an Alliance Futura analyzer.

### Bacterial abundance and production

Bacterial abundance was quantified by preserving 1.75 ml of seawater with 1% paraformaldehyde + 0.05% glutaraldehyde, freezing in liquid nitrogen, and storing at −80°C until analysis by flow cytometry (Beckman-Coulter® CytoFlex, CINBIO-University of Vigo facilities). Prokaryotic cells were stained with SYBR Green fluorochrome [57]. Bacterial production (BP) was measured using the ^3^H leucine incorporation method [58, 59], adding 40 μl of leucine (40 μCi ml^−1^) to 1-ml samples and incubating for 90 min. Leucine uptake was converted to BP using a carbon conversion factor of 3.1 kg C mol^−1^ Leu [59]. Please note that BP rates were not quantified at 24 and 48 h due to logistic constraints associated with radiolabeled compound work.

### Chlorophyll *a*

Chlorophyll *a* (Chl-*a*) concentration was measured as an estimate of phytoplankton biomass. Water samples (150 ml) were filtered (0.2 μm polycarbonate) and frozen at −20°C. Chl-*a* was extracted with 90% acetone and kept in darkness at 4°C overnight. Chl-*a* fluorescence was measured with a TD-700 Turner Designs fluorometer using a non-acidification technique [60] and an absorption coefficient of 87.7 at 663 nm [61].

### Primary production

Total primary production (PP) was measured by inoculating four 75-ml seawater samples (three with light and one dark) with 10 μCi NaH^14^CO₃ (370 kBq) and incubated under *in situ* conditions for 3 h. The samples were filtered (0.2 μm, 47 mm polycarbonate) under low vacuum pressure (<50 mmHg) and fumed overnight with HCl to remove unassimilated ^14^C. After adding 4 ml of scintillation cocktail, radioactivity in each sample was measured on a Wallac β-scintillation counter. Primary production was calculated by subtracting dark-bottle Disintegrations Per Minute (DPMs) from light-bottle DPMs [62]. Please note that PP rates were not quantified at 24 and 48 h due to logistic constraints associated with radiolabeled compound work.

### Metatranscriptomic analysis

For metatranscriptomic analysis, 2 l of seawater was taken in triplicate from the mesocosms at 0 and 72 h. The samples were filtered through 0.22-μm polyethersulfone membranes (Millipore® Sterivex™). Filters were filled with RNAlater, flash-frozen in liquid nitrogen, and stored at −80°C until further processing (time elapsed between inoculating the treatments and flash-freezing samples in liquid nitrogen was 2–3 h). Prokaryotic reads were filtered *in silico* from ribosomal-depleted non-selected RNA after taxonomic annotation (hereafter prokaryotic dataset), and the eukaryotic fraction present in the ribosomal-depleted dataset was filtered out. On the other hand, eukaryotic reads were sequenced from polyA-selected RNA (hereafter eukaryotic dataset). Extraction of total RNA was performed using the RNeasy Mini Kit (Qiagen), followed by DNase treatment with the TURBO DNA-free Kit (Invitrogen, Thermo Fisher Scientific). For prokaryotic mRNA, ribosomal RNA was removed from total RNA using the RiboMinus™ Bacteria Module Kit and the RiboMinus™ Concentration Module Kit, followed by mRNA linear amplification with the MessageAmp™ II-Bacteria Kit (Thermo Fisher Scientific). For eukaryotes, mRNA was isolated from total RNA using the Poly(A)Purist™ MAG Kit and subsequently linearly amplified with the MessageAmp™ II aRNA Kit (Thermo Fisher Scientific). For sequencing of the linearly amplified samples, TruSeq libraries were generated and sequenced at the Swedish National Genome Infrastructure, SciLifeLab Stockholm, using a HiSeq 2500 platform. One of the eukaryotic triplicates (R + P 0 h) was not included in the analyses due to insufficient sequencing depth, likely caused by RNA degradation or sample loss during processing. Therefore, the eukaryotic metatranscriptomic data for R + P 0 h comes from duplicates instead of triplicates (like the rest of the samples).

Raw reads were processed with the nf-core/metatdenovo pipeline (v.dev,1.0, commit 8e0d117; Di Leo *et al*. in prep.) built in Nextflow [63]. This pipeline performs the following steps: quality control using *FastQC* [64] and *MultiQC* [65]; primer sequence removal with *Cutadapt* [66] and sequence trimming with *Trim Galore*; *de novo* assembly with *Megahit* for both the prokaryote and eukaryote data [67]; open reading frame (ORF) determination with *Prokka* [68] for prokaryotic sequences and *Transdecoder* [69] for eukaryotic sequences; quantification using *BBmap* [70] and *FeatureCount* [71]; functional annotation with *EggNOG-mapper* [72, 73]; taxonomic assignment using *EUKulele* [74] against the Genome Taxonomy Database [75] for prokaryotes and MAR*MMETSP v2.0* for eukaryotes [76]. For prokaryotes, the metatdenovo analysis enabled the taxonomic assignment of 18% to 22% of the total reads to ORFs (nearly all to bacteria). Analysis of the polyadenylated RNA sequences enabled the classification of 25% to 31% of ORFs of eukaryotic taxa (Supplementary Table 2).

### Normalizations, statistics, and visualization

**Figure 2:**
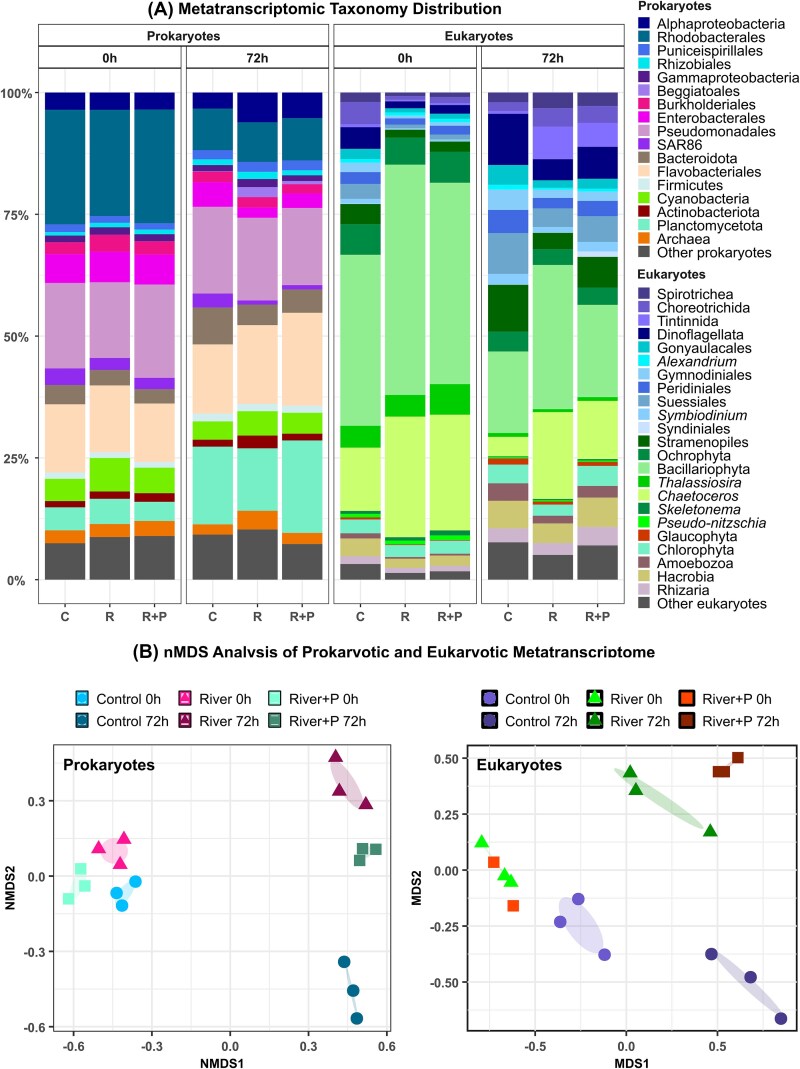
(A) Metatranscriptomic taxonomic distribution of prokaryotic (left) and eukaryotic (right) communities at 0 and 72 h in the three mesocosm treatments: C (*Control*), R (*River*), and R + P (*River + P*). Data from triplicates are shown for all treatments except for the *River + P* at 0 h which only includes duplicates. Taxonomic groups are shown as relative abundance percentages. (B) Non-metric multidimensional scaling (nMDS) analysis of prokaryotic and eukaryotic metatranscriptomes based on taxonomic composition and functional profiles across treatments and time points.

**Figure 3:**
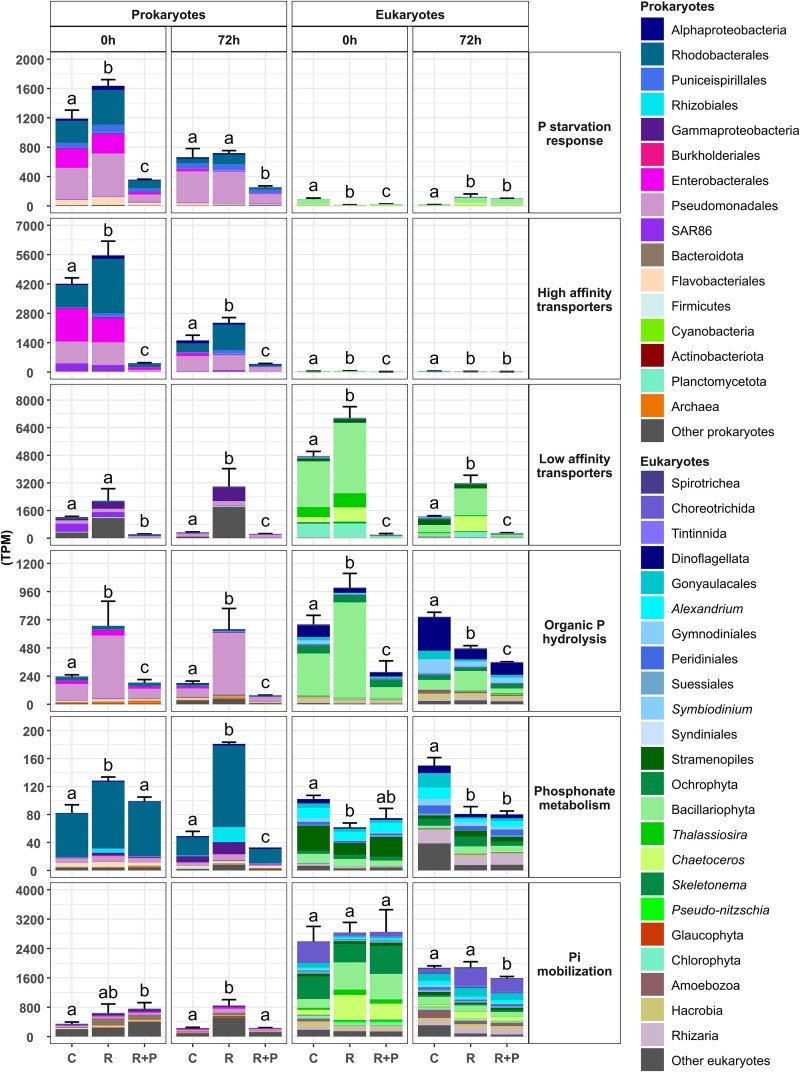
Relative abundance of transcripts (TPM) associated with phosphorus (P) metabolism in prokaryotic and eukaryotic communities at 0 and 72 h in the three mesocosm treatments: C (*Control*), R (*River*), and R + P (*River + P*). Bars represent the mean TPM values from biological replicates per treatment. Data from triplicates are shown for all treatments except for the *River + P* at 0 h which only includes duplicates. Error bars indicate the standard error of the mean. Statistical analysis was performed using a *t*-test with sample size–adjusted Good’s correction ($P\ast \surd n/100$) based on the mean total sum of transcripts without considering taxonomic composition. Different letters denote statistically significant differences between treatments.

**Figure 4:**
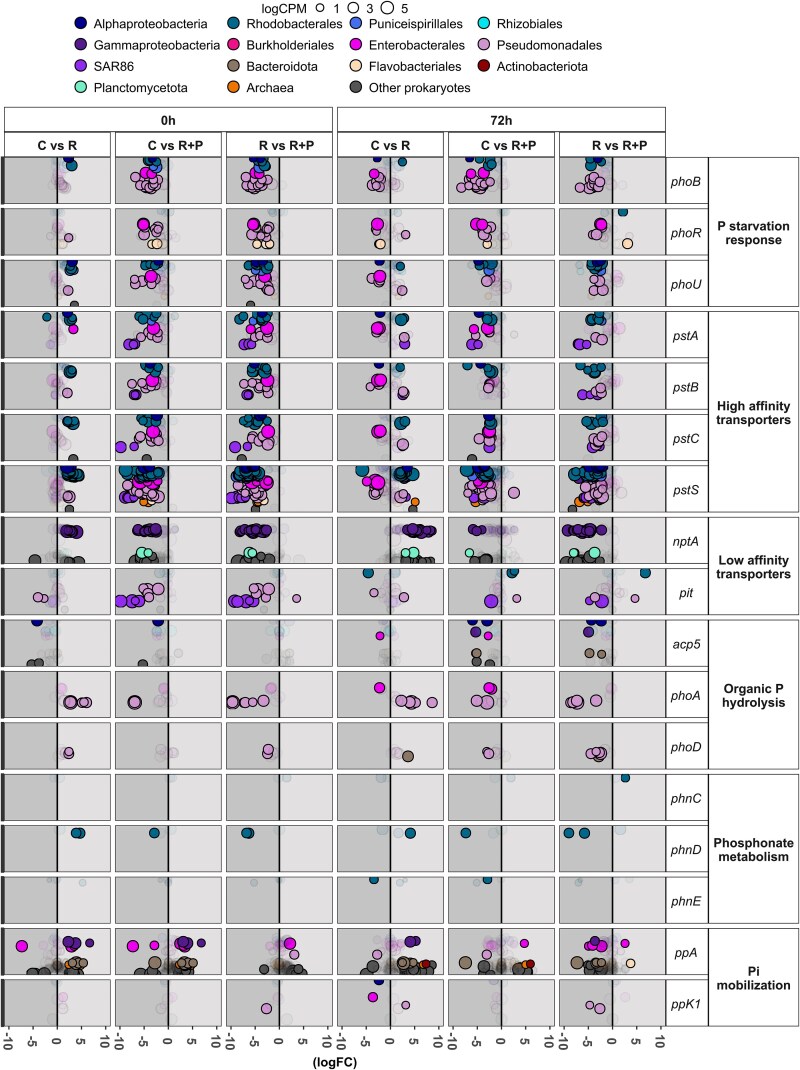
Differential gene expression of selected phosphate-related genes expressed by major prokaryotic groups in response to riverine additions. Bubble size corresponds to the mean (data from triplicates are shown for all treatments except for the *River + P* at 0 h which only includes duplicates) of the relative abundance (logCPM) of each gene. The position along the *y*-axis represents individual gene-level expression. Highly colored bubbles indicate significantly up- or downregulated genes (FDR < 0.05, *P* < .05, logCPM > 1, |logFC| > 2), whereas faded bubbles represent genes without significant differential expression (FDR > 0.05, *P* > .05, logCPM > 1, |logFC| > 2).

**Figure 5:**
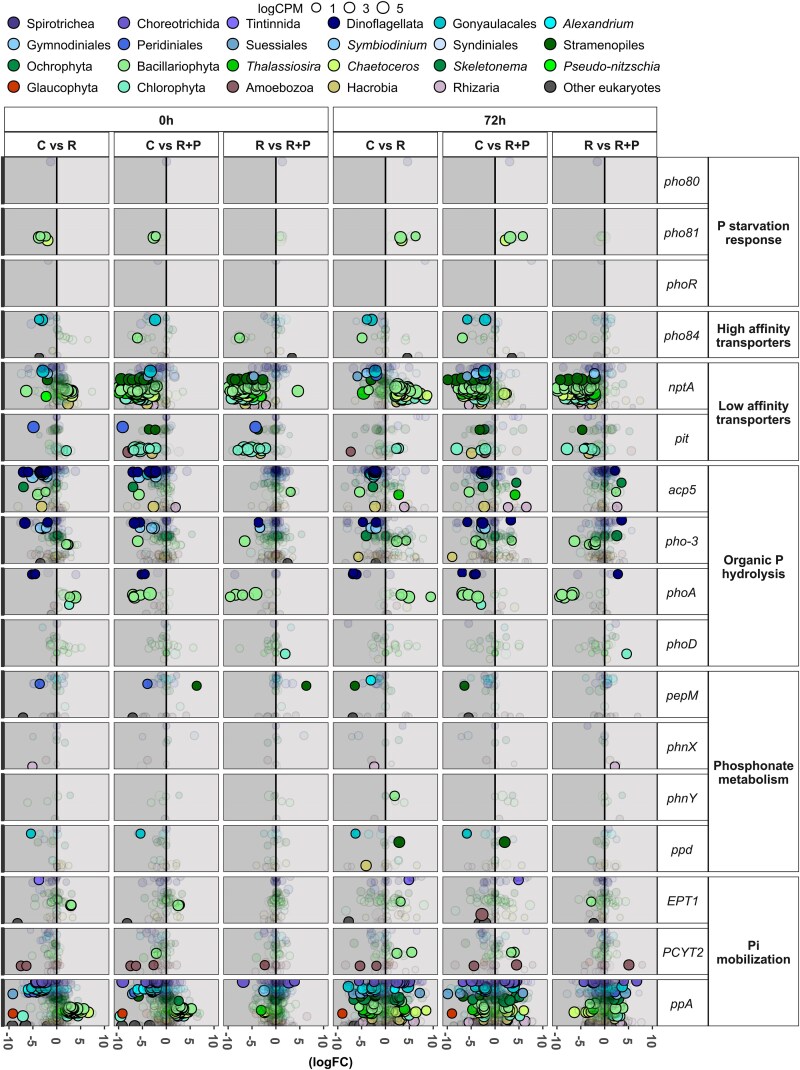
Transcriptional changes of selected phosphate-related genes expressed by major eukaryotic groups in response to riverine additions. Bubble size corresponds to the mean (data from triplicates are shown for all treatments except for the *River + P* at 0 h which only includes duplicates) of the relative abundance (logCPM) of each gene. The position along the *x*-axis represents individual gene-level expression. Highly colored bubbles indicate significantly up- or downregulated genes (FDR < 0.05, *P* < .05, logCPM > 1, |logFC| > 2), whereas faded bubbles represent genes without significant differential expression (FDR > 0.05, *P* > .05, logCPM > 1, |logFC| > 2).

To account for differences in transcript lengths and library sizes for comparative analysis between samples, for prokaryote data, we applied a transcript per million (TPM) normalization of reads mapping to individual prokaryote ORFs to the total of reads mapping to prokaryote ORFs. For the eukaryote data, TPMs for individual ORFs were normalized to the total of reads mapping to eukaryote ORFs (Fig. 2). Transcriptional similarity patterns were assessed using non-metric multidimensional scaling (nMDS) based on Bray–Curtis distances calculated from normalized TPM values. The analysis was performed with the *ggplot2* (v 3.4.1) [77] and *ggord* (v 1.1.7) [78] packages in R. Permutational multivariate analysis of variance with 999 permutations was carried out to test for significant differences between treatments using the *vegan* (v 2.6.4) package [79] (Fig. 2B). To evaluate the microbial activity related to phosphorus metabolism, we performed *t*-tests (*P* < .05) to compare the overall functional categories (Supplementary Table 1 and Fig. 3). Additionally, we conducted a differential gene expression (DGE) analysis using *edgeR* [80]. We focus our investigation on marker genes associated with different processes and pathways related to phosphorus metabolism. Individual genes with FDR <0.05 were considered differentially expressed (Figs. 4 and 5). When comparing differences of the sum of TPMs per category between treatments, *P* values were corrected using a sample size–adjusted Good’s correction (P*√*n*/100) [81]. Note that statistics are based on technical rather than biological triplicates (duplicates in the case of eukaryotic metatranscriptome in treatment R + P at 0 h).

## Results

### Hydrographic conditions and microbial dynamics in the mesocosms

#### Nutrient concentrations

The addition of riverine water in the *River* (*R*, P-deplete) and *River + P* (*R + P*, P-replete) treatments gave elevated initial concentrations of NO_3_^−^ and SiO_2_ which then rapidly decreased over time (Fig. 1B). The *R + P* addition increased initial PO₄^3−^ values up to 1.55 μmol l^−1^, which progressively decreased down to 0.24 μmol l^−1^ at the end of the experiment. PO₄^3−^ concentration remained below 0.2 μmol l^−1^ throughout the experiment in the *Control* (*C*, P-deplete) mesocosm and the *R* treatment. The N:P ratio in the *C* mesocosm increased from 5 at the beginning of the experiment to 25 at the end (Fig. 1B). By contrast, the initial N:P ratio in the *R* addition treatment was 49 and sharply decreased after 24 h down to 4.5 while the N:P ratio in the *R + P* treatments remained relatively low (below 5.5) throughout the experiment (Fig. 1B). NH_4_^+^ concentrations increased throughout the experiments in all mesocosms (Fig. 1B).

#### Microbial abundance and production

Bacterial abundance and production rates presented decreasing patterns in the control and in the addition treatments from initial values of ~3.35 × 10^6^ cells ml^−1^ and 37.7 μg C l^−1^ day^−1^, respectively (Fig. 1B). Similarly, the initial Chl-*a* concentration in the *C* mesocosm (1.61 μg l^−1^) progressively decreased until the end of the experiment. By contrast, in the *R* and *R + P* addition treatments, a similar increase in Chl-*a* concentration at 24-h incubation was registered (up to 2.35 μg l^−1^) (Fig. 1B). PP rates decreased throughout the experiment, yet the rates were lower in the *C* than in the *R* and *R + P* addition treatments at the end (Fig. 1B).

### Metatranscriptome taxonomic composition

The taxonomic affiliation of expressed prokaryotic functional genes changed in all mesocosms after 72 h of incubation (Fig. 2A). The nMDS analysis of the metatranscriptomes revealed a grouping of samples based on treatment and time (Fig. 2B). We observed an important decrease over time in the contribution of transcripts associated with Rhodobacterales (*P* > .05), Burkholderiales (*P* > .05), and Enterobacterales (*P* < .05) in all mesocosms. By contrast, transcripts affiliated to Plantomycetota, and to a lesser extent to Bacteroidota, showed a remarkable increase (*P* < .05) in all mesocosms. The eukaryotic transcription was dominated by Bacillariophyta (diatoms; mainly *Chaetoceros* and *Thalassiosira*), with a particularly high relative expression of *Chaetoceros* in the *R* and *R + P* treatments compared to *C* (*P* < .05) (Fig. 2A). We observed significantly higher (*P* < .05) relative expression of Dinophyceae (mainly Suessiales) and Spirotrichea (mainly Choreotrichida) in the *C* treatment than in the *R* and *R + P* treatments. After 72 h, the relative expression of Bacillariophyta significantly decreased (*P* < .05) while that of Dinophyceae increased (*P* < .05) in all treatments, particularly in the *C* mesocosm. The relative expression of *Tintinnida* significantly increased (*P* < .05) in the *R* and *R + P* treatments (Fig. 2A).

### Response of microbial communities to P availability

Overall, the transcriptional analyses showed that different taxonomic groups of both prokaryotes and eukaryotes contributed differently to the transcription of genes belonging to distinct P metabolic routes (Fig. 3). Furthermore, the relative expression of P-metabolism genes increased and decreased in the *R* and *R + P* treatments, respectively, compared to *C* at time 0 h (i.e. 2–3 h after the additions; see Fig. 3 for bulk changes and Figs. 4 and 5 for specific changes). Under P-limiting conditions, prokaryotes mostly transcribed genes encoding high-affinity transporters and to a lesser extent genes related to P-starvation response, low-affinity transporters, and organic P-hydrolysis. By contrast, eukaryotes mostly transcribed genes encoding low-affinity transporters and to a lesser extent genes related to organic P-hydrolysis. Genes related to internal Pi mobilization were mostly expressed by eukaryotes and seemed to be independent of Pi availability (Fig. 3).

### Transcriptional responses in prokaryotic P metabolisms

The relative transcription of genes associated with high-affinity phosphate transport in prokaryotes was higher than that of any other category studied. *C* and *R* accounted for 51–58% and 31–52% of total P metabolism–related transcripts, respectively (Supplementary Fig. 1). In *R + P*, although high-affinity transport genes still represented a substantial proportion (22–32%), Pi-mobilization genes exhibited the highest transcription levels (19–37%) (Supplementary Fig. 1). Genes encoding high-affinity transporters (phosphate-specific transport system, *pstABCS*, among others) and involved in P-starvation responses (Pho regulon, *phoBRU*, among others) were mainly transcribed by Rhodobacterales, Enterobacterales, Pseudomonadales, and SAR86 and followed a similar pattern. Their expression was significantly higher in *R* and lower in *R + P* compared to *C* (*P* < .05) at both 0 and 72 h (Fig. 3).

A similar, but less pronounced, response was observed in the expression of genes encoding low-affinity phosphate transporters. These included the sodium-dependent phosphate cotransporter *nptA* (primarily expressed by Gammaproteobacteria and Planctomycetota) and the phosphate inorganic transport system gene *pit* (mostly by SAR86 and Pseudomonadales), which showed varying transcription levels. At time 0, their relative expression was significantly higher in the *R* and *C* treatments compared to that in *R + P* (*P* < .05). After 72 h, these differences became more evident (*P* < .05; Fig. 3): expression of low-affinity transporter genes decreased in *C*, increased in *R*, and remained low in *R + P*. In general, transcripts associated with low-affinity phosphate transporters represented a smaller proportion of the total phosphorus metabolism–related expression compared to those associated with high-affinity transporters (10% to 38% depending on the treatment; Supplementary Fig. 1).

The relative transcription of genes related to phosphonate metabolism (e.g. the phosphonate transport system genes *phnCDE* by Rhodobacterales) and organic P-hydrolysis (e.g. alkaline phosphatases *phoAD* by Pseudomonadales and the acid phosphatase *acp5* by Bacteroidota and Gammaproteobacteria) was lower than that of other P-metabolism categories. Only 1.1%–4.7% and 3.2%–8.8% of the total transcripts in the phosphonate metabolism and organic P-hydrolysis categories, respectively, were detected across different treatments (Supplementary Fig. 1). However, the transcription of phosphonate metabolism significantly (*P* < .05) increased in *R* compared to that in the *C* and the *R + P* treatments at 0 and 72 h (Fig. 3). In contrast, the transcription of Pi-mobilization genes for internal polyP pools followed a distinct pattern. It significantly (*P* < .05) increased *R* after 72-h incubation while remaining low in *C* and decreasing in *R + P* at 72 h (Fig. 3).

The results of the DGE analysis (Fig. 4) were consistent with the trends observed in Fig. 3. Significant changes in relative expression were detected among prokaryotes. For example, genes associated with phosphorus starvation (*phoB*, *phoR*, *phoU*) and high-affinity transport (*pstA*, *pstB*, *pstC*, *pstS*) exhibited differential expression among all treatments, with a positive response compared to the control in the *R* treatment (P-depleted) and a negative response in *R + P* treatment (P-replete) (FDR < 0.05, *P* < .05, logCPM > 1, |logFC| > 2) (Fig. 4).

#### Response in eukaryotic transcription of P-metabolism genes

Unlike prokaryotes, the transcription of genes associated with high-affinity phosphate transport in eukaryotes accounted for only 1%–4% of total phosphorus metabolism–related transcripts (Supplementary Fig. 1). The relative transcription of genes associated with low-affinity transport was higher (*P* < .05) than that of any other category studied in *C* and *R*, representing 30–57% and 54–62%, respectively, of total P metabolism–related transcripts. In *R + P*, while low-affinity transport genes accounted for only 6–12%, Pi mobilization genes exhibited the highest transcription levels, reaching 25–83% of P-metabolism transcription. A strong and significant (*P* < .05) increase in the expression of genes encoding low-affinity phosphate transporters (e.g. *nptA* and *pit* genes) by different taxa within Bacillariophyta (e.g. *Thalassiosira*, *Chaetoceros*) and Dinoflagellata (e.g. Peridiniales, Gymnodiniales, *Symbiodinium*, Suessiales) among others was observed immediately (0 h) and also after 72 h in the treatments with a relatively higher N:P ratio (*C* and *R*) compared with *R + P* (Fig. 3).

Similarly, although to a lesser extent, a response was observed in the genes involved in organic P-hydrolysis (phosphatases *acp5*, *pho-3*, and *phoA*), which accounted for 8%–18% of the total of transcripts related to P acquisition in the different addition treatments (Fig. 3, Supplementary Fig. 1). These genes were mostly transcribed by Bacillariophyta (e.g. *Thalassiosira*) and Dinoflagellata (e.g. Gonyaulacales, Gymnodiniales, Suessiales), showing a significant (*P* < .05) increase or decrease immediately after P-deplete or P-replete riverine inputs (0 h; Fig. 3). By contrast, the expression of genes related to phosphonate metabolism (e.g. phosphoenolpyruvate phosphomutase *pepM* and phosphonopyruvate decarboxylase *ppD*) was relatively low (0.5%–3.6% of the total of transcripts related to P acquisition) and decreased (*P* < .05) in both *R* and *R + P* compared to *C* (Fig. 3, Supplementary Fig. 1). Finally, the relative transcription by eukaryotes of genes related to Pi mobilization from internal polyP pools was maintained elevated after the additions, although a significant decrease occurred in the *R + P* addition treatment (P-replete conditions) after 72 h (Fig. 3).

The results of the DGE analysis (Fig. 5) confirmed the trends observed in Fig. 3, with significant changes in the expression detected among eukaryotes. For example, genes associated with low-affinity transporters (*nptA* and *pit*) and organic P-hydrolysis (*acp5*, *pho-3*, and *phoA*) exhibited differential expressions in all treatments, with an immediate (0 h) negative response in the *R + P* treatment (P-replete) compared to both the C and R treatments (P-deplete). The relative transcription by eukaryotes of genes related to Pi mobilization from internal P pools followed different patterns in different taxonomic groups after the additions (Fig. 5). For example, at 0 h the transcription of pyrophosphate (PPi) mobilization genes by dinoflagellates, such as Gonyaucales, *Alexandrium,* or Gymnodiniales (e.g. *ppA*), significantly increased in *C* compared to *R* and *R + P* while the transcription by diatoms followed the opposite pattern and increased after the additions. After 72-h incubation, different dinoflagellate and diatom taxa significantly increased and decreased, respectively, the transcription of the inorganic pyrophosphatase *ppA* (Fig. 5) (FDR < 0.05, *P* < .05, logCPM > 1, |logFC| > 2).

#### Correlations between transcripts and environmental factors

**Figure 6:**
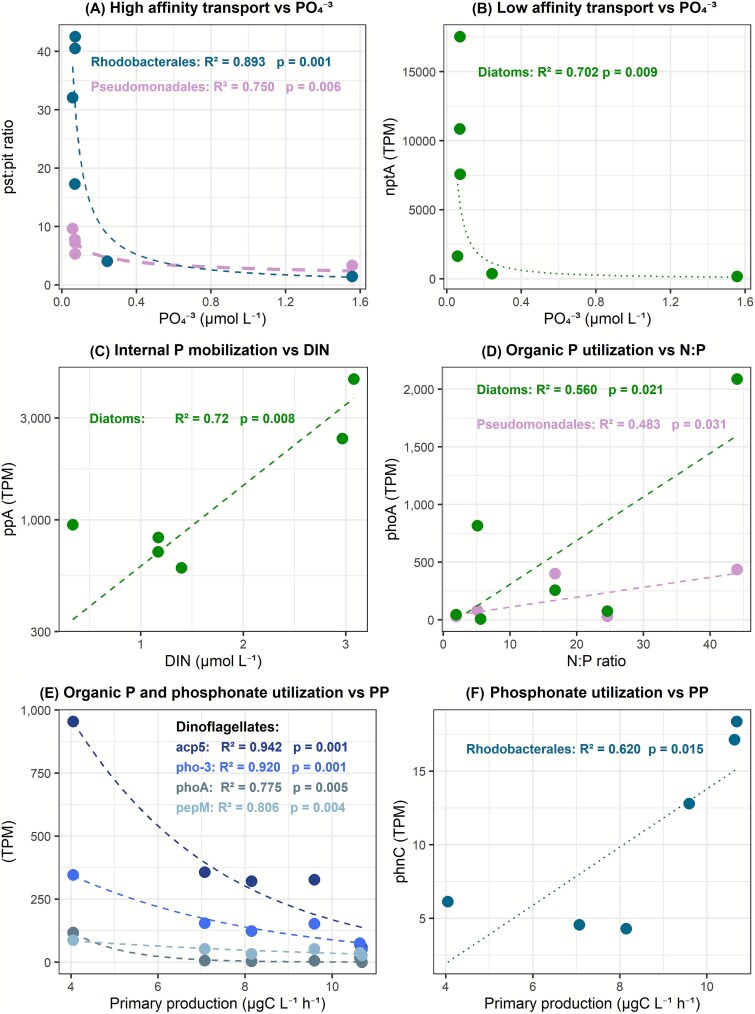
Correlation between the relative abundance of selected genes involved in phosphate metabolism and environmental variables across mesocosm treatments. A) Relationship between the *pst*/*pit* gene expression ratio and PO₄^3−^ concentration, modeled using a power regression; B-F) Correlations between gene expression levels (TPM) os specific genes and environmental variables, fitted with exponential regression models. The strength of these relationships is indicated by the coefficient of determination (*R*^2^) and *P* values obtained from the regression analyses corrected using a sample size–adjusted Good’s correction.

In order to provide support for specific results from the general patterns exposed in the previous sections, regression analyses between selected transcripts and environmental variables were performed. The ratio between the relative expression of *pst* and *pit* (used to highlight the different magnitude of the expression of both genes which are markers of high- and low-affinity P transport, respectively) in *Rhodobacterales* (*r*^2^ = 0.89) and Pseudomonadales (*r*^2^ = 0.75) was negatively correlated (*P* < .05) with phosphate concentration (Fig. 6). The relative abundance of *phoA* transcripts in Pseudomonadales (*r*^2^ = 0.48) and *phnC* in Rhodobacterales (*r*^2^ = 0.62) was positively correlated (*P* < .05) with N:P ratios and PP rates, respectively.

The relative abundance of diatom *phoA* (*r*^2^ = 0.56) and *nptA* (*r*^2^ = 0.70) transcripts was positively correlated (*P* < .05) with N:P ratios and phosphate concentration (Fig. 6). On the other hand, the relative abundance of *ppA* transcripts in diatoms (*r*^2^ = 0.72) was positively related (*P* < .05) with DIN concentration. Dinoflagellate *acp5*, *pho-3*, *phoA*, and *pepM* transcripts were negatively related (*P* < .05) with primary production (*r*^2^ = 0.94, *r*^2^ = 0.92, *r*^2^ = 0.77, and *r*^2^ = 0.81, respectively) (Fig. 6).

## Discussion

The present study offers new insights into the adaptation mechanisms used by natural microbial communities to acquire phosphorus (P) in coastal systems influenced by P-limited continental runoff. Two distinct transcriptional patterns were identified after riverine inputs, pinpointing specific microbial strategies in response to variations in nutrient availability. First, as could be expected, both eukaryotes (essentially photosynthetic protists, hereafter referred as phytoplankton) and prokaryotes (mainly heterotrophic bacteria, referred to as bacteria) increased their transcriptional investment into P uptake when subjected to P-limiting riverine discharge; upon P-replete input, sharp decreases in P-related transcription were instead recorded. Second, and particularly important, our results suggest that high N:P ratios associated with riverine discharge into coastal ecosystems may exacerbate competition for available P of microbial communities and force P niche-partitioning among microbial taxa. Interestingly, different bacterial and phytoplankton taxa adopted contrasting P-acquisition strategies under P-deplete conditions. Bacteria increased the transcription of genes encoding high-affinity phosphate transporters (with low uptake velocities) while phytoplankton preferentially transcribed genes encoding low-affinity phosphate transporters (with high uptake velocities), thus highlighting different trade-offs between binding strength and speed. Furthermore, our data indicated that different P-acquisition strategies play a key role in species succession in the phytoplankton communities (with diatoms quickly and efficiently utilizing phosphate at the beginning of blooms and dinoflagellates using DOP during bloom decay), whereas different bacterial taxa specialized on the utilization of distinct compounds within the DOP pool (easily accessible phosphate esters versus relatively challenging to metabolize phosphonates). Thus, the present dataset exemplifies functional mechanisms by which P availability plays a key role on phytoplankton and bacterial community structure and functioning in productive coastal systems.

### Microbial response to the riverine inputs

As shown in this and previous works [12, 52], riverine inputs in the Ría de Vigo not only increase environmental N:P ratios but also promote the growth of phytoplankton communities, particularly diatoms, due to the high concentration of nitrate and silicate associated with freshwater discharge. Teira *et al*. [12] demonstrated that while phytoplankton linearly respond to increasing amounts of riverine inputs in the Ría de Vigo, bacteria show the opposite trend and decrease in abundance, probably reflecting the ability of phytoplankton to outcompete bacteria for P uptake when nitrate concentration is high. This is consistent with the fact that, contrary to phytoplankton, bacteria are more commonly limited by P than N in marine coastal ecosystems [14, 82–85]. Even though previous studies showed that bacteria should be better competitors for P than phytoplankton under low P load [86], bacteria in the present study were not able to outcompete phytoplankton neither in P-deplete nor P-replete treatments, which strongly suggests that bacteria experienced limitation by bioavailable C, as consistently found in previous experimental studies in the area [20, 83, 87]. We find it plausible that this primary C limitation affects the ability of bacteria to successfully compete with phytoplankton under P-limiting conditions. Future work could be directed to compare P-acquisition strategies between phytoplankton and bacteria under C-replete conditions by experimentally adding a labile C source. It is also important to note that the general decrease observed in phytoplankton and the bacterial abundance throughout the experiment suggest a possible grazing pressure that is supported by the increase in relative abundance of Tintinnids which are extensively known as grazers of small phytoplankton and bacteria [88].

### Different inorganic P-acquisition strategies in bacteria and phytoplankton

Bacterial transcription of high-affinity transporters was particularly notable for e.g. Rhodobacterales (mostly *Amylibacter* and *Planktomarina*), Pseudomonadales (mostly *Porticoccaceae*), and SAR86 which transcribed genes that encode both the permeases (*pstABC*) and the phosphate-binding protein (*pstS*) of the transport system for orthophosphate. The high-affinity transport system for orthophosphate (*pstABCS*) plays an important role in inorganic phosphate uptake under P-limiting conditions in the Ría de Vigo [89], in the northwestern North Atlantic, and in subtropical to sub-Antarctic waters [90, 91]. This is related to the kinetics of the gene encoding the phosphate-binding protein (*pstS*) which is highly efficient under low Pi concentration but exhibits low uptake velocities [27]. In fact, the expression of the *pstS* of the transport system is a common indicator of microbial P stress, as shown in seasonal studies of *pstS* expression [41, 92–94]. Interestingly, in the present study, the relative bacterial transcription of genes for high-affinity transporters was high even in *C*, where a N:P ratio of ~5 indicated N limitation. However, P concentrations in *C* were low, suggesting that bacterial gene expression is tightly related to the absolute concentration of P (Fig. 6A).

In contrast to bacteria, the responsive phytoplankton (e.g. Bacillariophyta, Dinoflagellata) increased the relative transcription of low-affinity transporters (e.g. *pit*, *nptA*) in the *R* treatment (with decreased P availability) (Fig. 6B). Curiously, this is counter to the general recognition that the *pit* gene is a widespread low-affinity phosphate membrane transporter typically expressed under high phosphorus concentrations and post-bloom conditions [21]. Still, it confirms previous results from phytoplankton culture research [22, 47] and also from field studies in shelf waters in this coastal system [89] which have reported high *pit* gene expression by both phytoplankton and bacteria under low phosphate conditions. We acknowledge that it is also possible that Pi concentrations in the experiment (i.e. 0.07 μM) were not low enough to activate high-affinity transporters in eukaryotes, although this seems not very probable since previous studies have shown that the expression of high-affinity phosphate transporters and permeases in Haptophyta and Chlorophyta cultures is activated below 1 μM [23, 24]. It is generally recognized that in coastal productive systems such as Ría de Vigo, fluctuations in nutrient availability are frequently associated with upwelling dynamics or riverine discharge episodes. In such conditions, phytoplankton species utilization of low-affinity transporters allows quick uptake that is related to the kinetics of the *pit* gene which is not efficient under low Pi concentration but exhibits high uptake velocities [27] and storage of Pi when in excess [3]. The storage of Pi as internal polyphosphate is a common strategy used by large cells (i.e. diatoms and dinoflagellates in the present work) to cope with P limitation as they may use the stored P during periods of P shortage [22], which likely explains that the relative importance of this process was higher in phytoplankton than in bacteria. Interestingly, our results show that both bacterial and phytoplankton communities may regulate the relative transcription of these genes after riverine inputs attending to P availability, suggesting that both microbial compartments eventually rely on the mobilization of stored Pi during P-limitation periods in the Ría de Vigo. Unexpectedly, diatoms highly expressed the *ppA* gene involved in pyrophosphate (PPi) hydrolysis immediately after both P-deplete and P-replete riverine additions. Thus, it seems that after P-replete riverine additions, diatoms may be mobilizing internal Pi reserves regardless of the high ambient Pi availability. This is consistent with findings by Diaz *et al*. [95], who suggested that PPi degradation in diatoms may persist irrespective of prevailing P levels. This suggests that eventually the mobilization of internal Pi reserves when N is available (as in the riverine inputs) (Fig. 6C) could be energetically more favorable than the expression of Pi transporters.

### Organic phosphorus utilization

P may be associated with C and N in a variety of organic compounds, including monoesters, diesters, nucleotides, nucleic acids, phosphonates, and phospholipids that are released by different biological processes such as exudation or cell lysis. Microbial plankton is well adapted to use organic P forms [96–99]. In fact, eukaryotic phytoplankton (e.g. dinoflagellates) have been shown to downregulate the expression of genes responsible for both low- and high-affinity phosphorus transport under P-deficient conditions while upregulating genes involved in the use of DOP [100]. Thus, in chronically P-limited marine ecosystems such as the Mediterranean Sea or the North Pacific Ocean, the genes encoding alkaline phosphatases (to hydrolyze phosphate esters) or C–P lyases (to extract P from phosphonates) contribute up to 30% and 10%, respectively, to the total abundance of P-acquisition genes in bacterial metagenomes [46, 101]. By contrast, the upwelling area of Ría de Vigo is not a long-term P-limited ecosystem since it intermittently receives inorganic phosphate inputs. Thus, the relative importance of transcripts related to organic phosphorus utilization was lower than that of genes related to inorganic P transport, suggesting that microbes in this system may invest more energy to utilize inorganic versus organic P forms. However, the present dataset suggests that both bacteria and phytoplankton may utilize organic phosphorus in the Ría de Vigo and that different patterns of response to riverine inputs were observed depending on the organic compound utilized and the taxa involved.

Our results suggest that the utilization of organic phosphorus by microbial communities was related to a low availability of P compared to N. As an example, diatoms, the ones that positively responded to the N-rich inputs and were likely responsible of the Chl-*a* maximum recorded at 24 h (Fig. 1B), importantly increased the relative transcription of genes in the intracellular phosphatase system (*phoA*), which is probably related to internal organophosphate hydrolysis, in response to Pi scarcity (Fig. 6D). Similarly, different prokaryotes, particularly Pseudomonadales (particularly *Cellvibrionaceae* and *Luminiphilus*), experienced an important relative increase in the transcription of genes in the phosphatase systems (*phoAD*) related to the decrease in P availability (Fig. 6D). The immediate increase in the expression of genes encoding phosphatases in the *R* treatment compared to that in the *C* (i.e. at 0 h) confirms that both phytoplanktonic and bacterial taxa took advantage of the availability of organic substrates following the experimental addition. Both microbial compartments shared this organic P-acquisition strategy, yet only bacteria maintained a very high level of phosphatases expression 72 h after the riverine inputs. This may be attributed to the fact that after 72 h, phytoplankton growth could have become nitrate limited.

Interestingly, riverine inputs increased the transcription of genes related with phosphonate utilization by bacteria but not phytoplankton, suggesting that utilization of phosphonates may be a strategy used during P-limiting conditions primarily by bacteria. Our data suggest that the relative increase in the utilization of phosphonates by Rhodobacterales (mostly *Amylibacter* and *Planktotalea*) is related to the utilization of phytoplankton-derived DOM and is in accordance with the role of this group as main DOM consumers during phytoplankton blooms [92, 102]. This result suggests that in the context of decreasing Pi availability (which will increase the relative contribution of organic P to the total available P pool), microbial communities in this system may be capable of responding by intensifying their transcriptional investment toward organic P utilization.

### Differential P-acquisition strategies within microbial compartments

The present dataset provides several examples of niche-partitioning between members of the two microbial compartments studied. On the one hand, differential P-acquisition strategies of phytoplankton taxa shown here highlight several functional traits that can play an important role in species succession in phytoplankton blooms in the Ría de Vigo. It is well known that during bloom progression and decay, nitrate and phosphate are consumed, organic nutrients (DOP and DON) accumulate due to processes like grazing and excretion by zooplankton and the release by phytoplankton and cell lysis [103], and subsequently remineralization leads to the availability of ammonium. These processes (i.e. diatom–dinoflagellate succession associated to changes in nutrient availability) were described in the conceptual framework of the “Margalef mandala” [104] and have been shown to promote a shift from a diatom-dominated community to a dinoflagellate-dominated community in the area of study [105–107] and in different environments around the global ocean (e.g. North Pacific [108, 109], North Atlantic [110, 111], Baltic Sea [112], and Mediterranean Sea [111, 113, 114]). In the present study, dinoflagellates became relatively more abundant in the metatranscriptomes after the Chl-*a* maximum and showed a clear tendency to then increase their transcription of genes related to the organic P hydrolysis, phosphonate utilization (Fig. 6E), and internal P mobilization, whereas the transcription of low-affinity Pi transporters remained largely dominated by diatoms. Thus, our results suggest that during the bloom decay, the relatively higher capability of DOP utilization of dinoflagellates compared with that of coexisting diatoms may allow dinoflagellates to be more competitive.

On the other hand, utilization of low-affinity transporters for Pi uptake may have helped diatoms to display high maximum growth rates when dissolved inorganic nutrients (e.g. Pi and DIN) are available (often in early successional stages). In addition to facilitating rapid nutrient uptake, low-affinity systems such as *pit* operate using the proton motive force, making them relatively energy efficient. In contrast, high-affinity systems such as *pst* are activated under phosphate limitation and rely on ATP hydrolysis, and are thereby more energetically costly. The use of *pit* at the beginning of blooms could, therefore, provide an advantage for diatoms in both uptake velocity and energy efficiency.

In the case of heterotrophic bacteria, our data highlight that different taxonomic groups specialize in the utilization of distinct P-containing organic compounds. Thus, the transcription of phosphatases was dominated by Pseudomonadales (particularly *Cellvibrionaceae*), suggesting that this group preferentially obtains P from phosphate esters (Fig. 6D) included in easily accessible compounds like nucleotides, nucleic acids, phospholipids, phosphoglycans, or phosphoproteins. In contrast to phosphate esters, phosphonates are relatively challenging to metabolize and can be found in the extracellular layers of polysaccharides produced by phytoplankton and bacteria to facilitate aggregation, defense, or biofilm formation [115]. It is therefore notable that Rhodobacterales (particularly *Amylibacter*) substantially contributed to the transcription of genes for the utilization of phosphonates, particularly when primary production rates increased (Fig. 6F) [116]. Both Pseudomonadales and Rhodobacterales grow associated to phytoplankton-derived organic matter, but they metabolize distinct N- and C-containing components of the DOM pool [117, 118]. It is also interesting to note that although the contribution of Rhodobacterales to the metatranscriptomes decreased after the Chl-*a* maximum, their contribution to the transcription of high-affinity Pi transporters and genes related to phosphonate utilization remained high, which confirms that this group importantly relies on both Pi and phosphonate utilization for P acquisition.

In conclusion, the present work highlights the mosaic of microbial processes governing P utilization by microbial communities in this coastal system subjected to P-limited riverine inputs. The knowledge that emerges from this work on the partitioning of different P compounds between distinct microbial taxa contributes to the understanding of resource utilization, competition, and species succession. Several genes appeared to be suitable indicators of the P availability or productivity of this dynamic coastal system, which may have application for monitoring purposes. Furthermore, unraveling the mechanisms utilized by microbial communities to cope with N-rich continental inputs and a higher proportion of organic P compounds is crucial in the context of future modeling attempts aiming to predict biogeochemical processes in productive ecosystems subjected to anthropogenically affected continental runoff.

## Supplementary Material

Supplementary_material_ycag035

## Data Availability

The datasets generated during and/or analyzed during the current study are available in the European Nucleotide Archive (ENA) repository (https://urldefense.com/v3/__https://www.ebi.ac.uk/ena/browser/view/PRJEB94162--;!! D9dNQwwGXtA!S9xqrzP3cWwgqoh8g9nDY5vabSsb21DHPdg1f3jf9153JR2qmi-MD6-mB3ZWXMDyeBLBjyK1touLCNl3WS5X8OHDRw$). Sample accessions are ERS25306826–ERS25306860 and sequence accessions are ERR15316847–ERR15316881.
