# Supplementary material for: Dealing with phosphorus deficiency: contrasting strategies in marine phytoplankton and bacteria
Source: ISME Commun. 2026 Feb 20;6(1):ycag035. doi: 10.1093/ismeco/ycag035 (PMC12981677; doi:10.1093/ismeco/ycag035)
Supplement: Supplementary_material_ycag035 [file supplementary_material_ycag035.zip › Supplementary Figures and Tables legends.docx]

# **Supplementary Figures legends**

**Supplementary Figure 1.** Relative abundance (%) of transcripts associated with phosphorus metabolism in prokaryotic (left) and eukaryotic (right) communities at 0 h and 72 h across the three mesocosm treatments: C (*Control*), R (*River*), and R+P (*River+P*). The bars represent the proportion of transcripts assigned to six functional categories relative to a curated set of 40 phosphorus-related genes.

# **Supplementary Tables legends**

**Supplementary Table 1.** Curated collection of genes involved in phosphorus metabolism. The table summarizes key genes grouped by functional subcategories and associated with a KEGG orthologies identifier (KOs). This collection integrates findings from relevant bibliographic reference supporting its functional and ecological relevance.

**Supplementary Table 2.** Summary of metatranscriptomic sequencing data from procaryotes and eukaryotes from mesocosm samples across time and treatments.
