# Supplementary material for: Dealing with phosphorus deficiency: contrasting strategies in marine phytoplankton and bacteria
Source: ISME Commun. 2026 Feb 20;6(1):ycag035. doi: 10.1093/ismeco/ycag035 (PMC12981677; doi:10.1093/ismeco/ycag035)
Supplement: Supplementary_material_ycag035 [file supplementary_material_ycag035.zip › Supplementary methods.docx]

# **Supplementary methods**

## ***Experimental design***

To evaluate the interactions shaping microbial community responses to riverine inputs, we performed a mesocosm experiment during spring 2019, using three 500 L UV-stabilized polyethylene bags maintained under in situ conditions using a floating structure anchored nearby the Toralla Marine Science Station (ECIMAT) in the Ría de Vigo (42° 12' 7.081" N, 8° 47' 53.830" W, Figure 1a). Three treatments were established: The Control (C) mesocosm was filled with 500 L of surface seawater (filtered through 200 µm mesh) from the Ría de Vigo, representing natural P-deplete conditions. The second, River (R) mesocosm included 450 L of surface seawater and 50 L of riverine water (10%) collected 1 day before the addition upstream of the freshwater-seawater interface of the Oitabén River, to ensure the representativeness of the river's chemical composition, and filtered through a 200 µm mesh. The third mesocosm, River+P (R+P), contained the same mixture as River, with an additional phosphorus enrichment (1 µM final concentration) to simulate P-replete conditions. Previous studies support the use of 10% riverine input to test the response of the Ría de Vigo to fluvial contributions.

## ***Inorganic and organic nutrient determinations***

In brief, the concentration of dissolved organic carbon (DOC) and dissolved organic nitrogen (DON) was estimated using a Shimadzu TOC-V Total Organic C Analyzer equipped with a Shimadzu TNM-1 Total N Measurement Unit. Seawater aliquots of 50 ml were filtered by 0.2µm filters (Pall, Supor membrane Disc Filter) and collected into pre-combusted (450°C, 20 h) glass bottles. DON was calculated by subtracting nitrite + nitrate+ ammonium from total dissolved nitrogen (TDN).  The concentrations of nitrate (NO3-), ammonium (NH₄⁺), phosphate (PO₄³⁻), and silicate (SiO2H4) were estimated by taking seawater aliquots of 50 ml in polyethylene bottles using contamination-free plastic gloves before all other variables to avoid contamination. Samples were analyzed with an Alliance Futura segmented flow analyzer by standard colorimetric method (Brewer, 1978). We used the fluorometric method of Kérouel and Aminot [48] to measure ammonium concentration. The detection limit was 0.1 µmol L−1 for NO3-, 0.02 µmol L−1 for NO2- and PO₄³⁻, and 0.05 µmol L−1 for NH₄⁺ and SiO2- µmol L−1.
