# Supplementary material for: Dealing with phosphorus deficiency: contrasting strategies in marine phytoplankton and bacteria
Source: ISME Commun. 2026 Feb 20;6(1):ycag035. doi: 10.1093/ismeco/ycag035 (PMC12981677; doi:10.1093/ismeco/ycag035)
Supplement: Supplementary_material_ycag035 [file supplementary_material_ycag035.zip › Supplementary_Table_1.pdf]

Gene	Category	Subcategory	KEGG_ko	Reference			
pho84	Phosphorus metabolism	High affinity transporters	K08176	Ogawa et al. 2000			
pstA	Phosphorus metabolism	High affinity transporters	K02038	Harke, Gobler 2013	Dai et al. 2020	Fischer et al. 2006	
pstB	Phosphorus metabolism	High affinity transporters	K02036	Hu et al. 2023	Hu et al. 2023	Dai et al. 2020	Fischer et al. 2006
pstC	Phosphorus metabolism	High affinity transporters	K02037	Satinsky et al. 2017	Hu et al. 2023	Dai et al. 2020	Fischer et al. 2006
pstS	Phosphorus metabolism	High affinity transporters	K02040	Willis et al. 2019	Hu et al. 2023	Dai et al. 2020	Fischer et al. 2006
ugpA	Phosphorus metabolism	High affinity transporters	K05814	Luo et al. 2009	Hu et al. 2023	Hsieh, Wanner 2010	
ugpB	Phosphorus metabolism	High affinity transporters	K05813	Luo et al. 2009	Hu et al. 2023	Hsieh, Wanner 2010	
ugpC	Phosphorus metabolism	High affinity transporters	K05816	Luo et al. 2009	Hu et al. 2023	Hsieh, Wanner 2010	
ugpE	Phosphorus metabolism	High affinity transporters	K05815	Luo et al. 2009	Hu et al. 2023	Hsieh, Wanner 2010	
ugpQ	Phosphorus metabolism	High affinity transporters	K01126	Luo et al. 2009	Hu et al. 2023	Hsieh, Wanner 2010	
nptA	Phosphorus metabolism	Low affinity transporters	K14683	C.-Y. Shih et al 2015	Lebens et al. - 2002		
pho1	Phosphorus metabolism	Low affinity transporters	K24195	Prodhan et al. 2022	Stefanovic et al. 2007	Hamburger et al. 2002	Rezzonico et al. 2004
pit	Phosphorus metabolism	Low affinity transporters	K14640	Lin, Litaker, Sunda 2016	Hu et al. 2023	Dai et al. 2020	Zou et al. 2020
pit	Phosphorus metabolism	Low affinity transporters	K03306	Lin, Litaker, Sunda 2016	Hu et al. 2023	Dai et al. 2021	Zou et al. 2021
acp5	Phosphorus metabolism	Organic P hydrolysis	K14379	Xu et al. 2020			
appA	Phosphorus metabolism	Organic P hydrolysis	K01093	Xu et al. 2020	Hu et al. 2023		
pho	Phosphorus metabolism	Organic P hydrolysis	K01078	Xu et al. 2020			
pho-3	Phosphorus metabolism	Organic P hydrolysis	K22390	Qi et al. 2016			
phoA	Phosphorus metabolism	Organic P hydrolysis	K01077	Willis et al. 2019	Robidart et al. 2019		
phoD	Phosphorus metabolism	Organic P hydrolysis	K01113	Shilova et al. 2014	Lin, Litaker, Sunda 2016	Hu et al. 2023	
phoE	Phosphorus metabolism	Organic P hydrolysis	K15640	Hsieh, Wanner 2010			
phoN	Phosphorus metabolism	Organic P hydrolysis	K09474	Neal et al. - 2018	Hu et al. 2023		
PPX1	Phosphorus metabolism	Organic P hydrolysis	K01514	Xu et al. 2020	Lindner et al. 2009		
ppx-gppA	Phosphorus metabolism	Organic P hydrolysis	K01524	Lindner et al. 2009			
pho4	Phosphorus metabolism	P starvation response	K06658	Qi et al. 2016			
pho80	Phosphorus metabolism	P starvation response	K06654	Qi et al. 2016			
pho81	Phosphorus metabolism	P starvation response	K06653	Qi et al. 2016			
pho85	Phosphorus metabolism	P starvation response	K06655	Qi et al. 2016			
pho87	Phosphorus metabolism	P starvation response	K14430	Xu et al. 2020	Qi et al. 2016		
phoB	Phosphorus metabolism	P starvation response	K07657	Lin, Litaker, Sunda 2016	Hu et al. 2023		
phoH-phoL	Phosphorus metabolism	P starvation response	K06217	Shilova et al. 2014	Robidart et al. 2019	Martiny et al. 2006	
phoR	Phosphorus metabolism	P starvation response	K07636	Lin, Litaker, Sunda 2016	Hsieh, Wanner 2010		
phoU	Phosphorus metabolism	P starvation response	K02039	Shilova et al. 2014	Hu et al. 2023		
psiE	Phosphorus metabolism	P starvation response	K13256	Hsieh, Wanner 2010	Santos-Beneit 2015		

pepM	Phosphorus metabolism	Phosphonate metabolism	K01841	Acker et al. 2022			
phnA	Phosphorus metabolism	Phosphonate metabolism	K19670	Lockwood et al. 2022	Shilova et al. 2014	Hu et al. 2023	
phnC	Phosphorus metabolism	Phosphonate metabolism	K02041	Lockwood et al. 2022	Lin, Litaker, Sunda 2016	Hu et al. 2023	Dai et al. 2023
phnD	Phosphorus metabolism	Phosphonate metabolism	K02044	Lockwood et al. 2022	Lin, Litaker, Sunda 2016	Shilova et al. 2014	Hu et al. 2023
phnE	Phosphorus metabolism	Phosphonate metabolism	K02042	Lockwood et al. 2022	Lin, Litaker, Sunda 2016	Hu et al. 2023	Dai et al. 2023
phnF	Phosphorus metabolism	Phosphonate metabolism	K02043	Lockwood et al. 2022	Hu et al. 2023		
phnG	Phosphorus metabolism	Phosphonate metabolism	K06166	Lockwood et al. 2022	Hu et al. 2023		
phnH	Phosphorus metabolism	Phosphonate metabolism	K06165	Lockwood et al. 2022	Hu et al. 2023		
phnI	Phosphorus metabolism	Phosphonate metabolism	K06164	Lockwood et al. 2022	Hu et al. 2023		
phnJ	Phosphorus metabolism	Phosphonate metabolism	K06163	Lockwood et al. 2022	Shilova et al. 2014	Hu et al. 2023	
phnK	Phosphorus metabolism	Phosphonate metabolism	K05781	Lockwood et al. 2022	Hu et al. 2023		
phnL	Phosphorus metabolism	Phosphonate metabolism	K05780	Lockwood et al. 2022	Hu et al. 2023		
phnM	Phosphorus metabolism	Phosphonate metabolism	K06162	Lockwood et al. 2022	Hu et al. 2023		
phnN	Phosphorus metabolism	Phosphonate metabolism	K05774	Lockwood et al. 2022	Hu et al. 2023		
phnO	Phosphorus metabolism	Phosphonate metabolism	K09994	Lockwood et al. 2022	Hu et al. 2023		
phnP	Phosphorus metabolism	Phosphonate metabolism	K06167	Lockwood et al. 2022	Hu et al. 2023		
phnPP	Phosphorus metabolism	Phosphonate metabolism	K20859	Lockwood et al. 2022			
phnW	Phosphorus metabolism	Phosphonate metabolism	K03430	Lockwood et al. 2022	Acker et al. 2022	Hu et al. 2023	
phnX	Phosphorus metabolism	Phosphonate metabolism	K05306	Lockwood et al. 2022	Acker et al. 2022	Hu et al. 2023	
phnY	Phosphorus metabolism	Phosphonate metabolism	K00206	Lockwood et al. 2022	Acker et al. 2022		
phnY	Phosphorus metabolism	Phosphonate metabolism	K21195	Lockwood et al. 2022	Acker et al. 2022		
phnY	Phosphorus metabolism	Phosphonate metabolism	K23996	Lockwood et al. 2022	Acker et al. 2022		
phnZ	Phosphorus metabolism	Phosphonate metabolism	K21196	Lockwood et al. 2022	Acker et al. 2022		
phpC	Phosphorus metabolism	Phosphonate metabolism	K12904	Lockwood et al. 2022			
ppd	Phosphorus metabolism	Phosphonate metabolism	K09459	Acker et al. 2022	Jimenez-Infante et al. 2017	Villarreal-Chiu et al. 2012	
ptxD	Phosphorus metabolism	Phosphonate metabolism	K18916	Frischkorn et al. 2019	Martínez et al. 2012		
ppA	Phosphorus metabolism	Pi movilization	K01507	Gómez-García et al. 2003	Hu et al. 2023		
ppK1	Phosphorus metabolism	Pi movilization	K00937	Jin et al. 2023			
ppk2	Phosphorus metabolism	Pi movilization	K22468	Jin et al. 2023			
