# Supplementary material for: Dealing with phosphorus deficiency: contrasting strategies in marine phytoplankton and bacteria
Source: ISME Commun. 2026 Feb 20;6(1):ycag035. doi: 10.1093/ismeco/ycag035 (PMC12981677; doi:10.1093/ismeco/ycag035)
Supplement: Supplementary_material_ycag035 [file supplementary_material_ycag035.zip › Supplementary_Table_2.pdf]

TABLE S1 | Metatranscriptome data summaries for mesocosm samples

Time	0 hours									72 hours								
**Mesocosm**	**C**			**R**			**R+P**			**C**			**R**			**R+P**		
**Replicate**	**1**	**2**	**3**	**1**	**2**	**3**	**1**	**2**	**3**	**1**	**2**	**3**	**1**	**2**	**3**	**1**	**2**	**3**
**Prokaryotes**																		
**Raw reads (10^6)**	8.01	7.99	7.81	7.14	5.81	5.29	8.19	5.81	5.29	6.22	6.99	5.82	6.03	7.32	5.92	5.62	5.54	5.70
**Reads with taxonomical assignment (10^6)**	3.40	3.31	2.90	3.08	2.47	2.14	4.15	2.47	2.14	1.92	1.86	1.86	2.36	2.70	2.14	1.96	1.92	1.90
Percentage of reads annotated as:																		
**Eukaryotic**	0%	0%	0%	0%	0%	0%	0%	0%	0%	0%	0%	0%	0%	0%	0%	0%	0%	0%
**Archaea**	0.98%	1.04%	1.04%	1.41%	0.93%	0.89%	1.52%	0.93%	0.89%	0.54%	0.49%	0.70%	1.32%	1.73%	1.16%	0.77%	0.76%	0.73%
**Bacteria**	41.49%	40.37%	36.12%	41.66%	41.56%	39.54%	49.23%	41.56%	39.54%	30.35%	26.16%	31.33%	37.90%	35.11%	35.03%	34.08%	33.97%	32.66%
Unidentified	57.53%	58.58%	62.84%	56.93%	57.51%	59.57%	49.25%	57.51%	59.57%	69.11%	73.35%	67.97%	60.78%	63.16%	63.81%	65.15%	65.27%	66.61%
**Eukaryotes**																		
**Raw reads (10^6)**	21,9	29,3	28,6	32,9	24,8	26,3	27,1E	28,3	-	27,8	21,4	26,2	29,5	28,4	24,4	17,1	23,1	24,2
**Reads with taxonomical assignment (10^6)**	7,02	1,00	9,55	12,1	8,81	9,45	9,48	10,3	-	7,54	5,62	7,54	9,80	9,60	7,49	4,93	6,70	7,02
Percentage of reads annotated as:																		
**Eukaryotic**	31,70%	33,88%	33,14%	36,63%	35,19%	35,72%	34,67%	36,26%	-	26,65%	25,77%	28,44%	33,03%	33,59%	30,37%	28,51%	28,53%	28,61%
**Archaea**	0,02%	0,01%	0,01%	0,00%	0,00%	0,00%	0,00%	0,00%	-	0,04%	0,03%	0,02%	0,01%	0,01%	0,02%	0,04%	0,03%	0,03%
**Bacteria**	0,35%	0,27%	0,29%	0,12%	0,25%	0,19%	0,25%	0,11%	-	0,48%	0,46%	0,33%	0,16%	0,18%	0,29%	0,39%	0,36%	0,37%
Unidentified	67,93%	65,84%	66,56%	63,25%	64,56%	64,09%	65,08%	63,63%	-	72,84%	73,74%	71,21%	66,79%	66,22%	69,32%	71,07%	71,07%	70,99%

*Sequence data represents the 3-0.22 µm size fraction. *Open reading frames (ORFs) of both Eukaryotes and Prokaryotes.*
