# Supplementary figures and images for: Dealing with phosphorus deficiency: contrasting strategies in marine phytoplankton and bacteria
Source: ISME Commun. 2026 Feb 20;6(1):ycag035. doi: 10.1093/ismeco/ycag035 (PMC12981677; doi:10.1093/ismeco/ycag035)

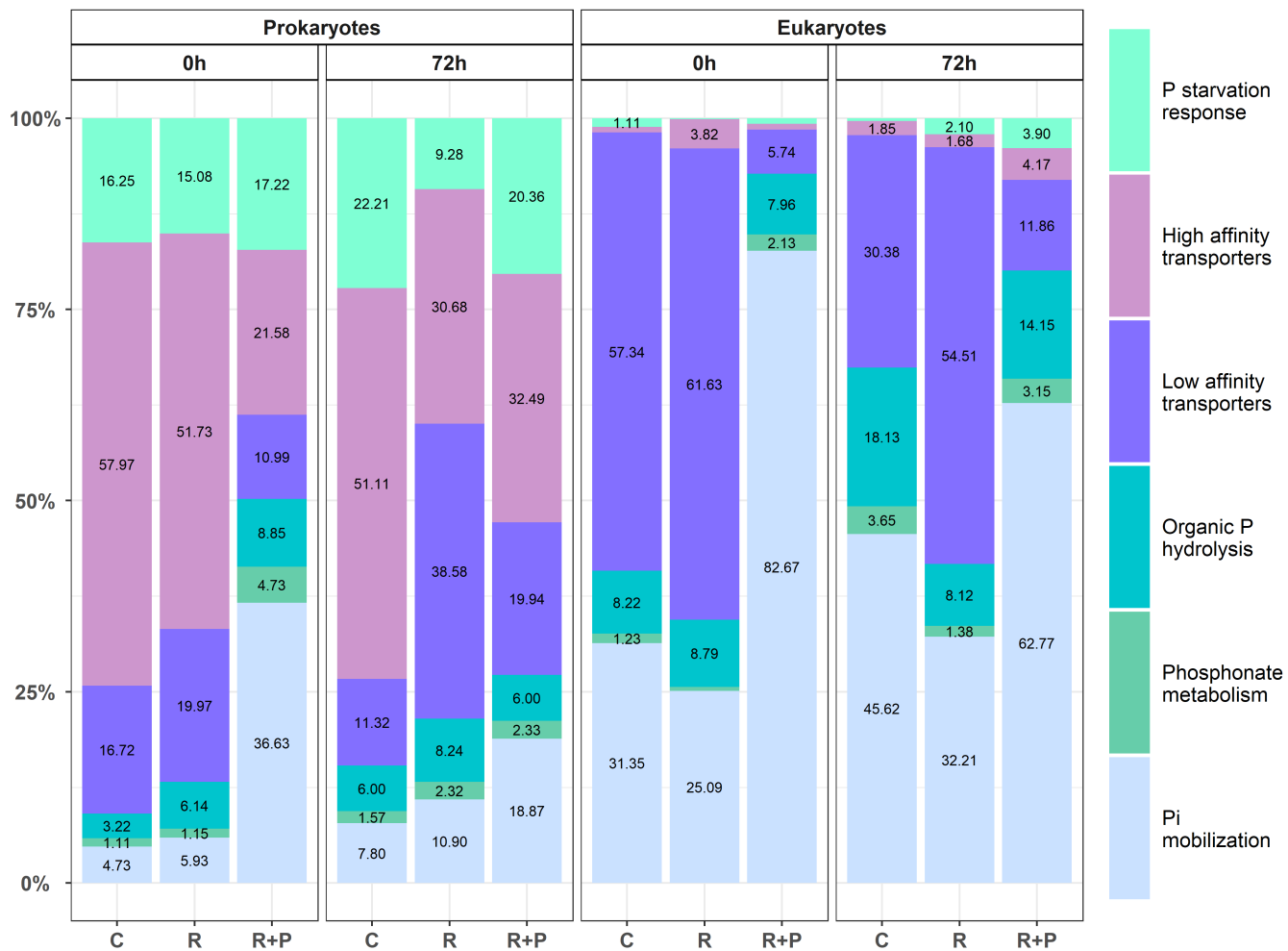

Supplement: Supplementary_material_ycag035 [file supplementary_material_ycag035.zip › Supplementary_Figure_1.pdf]
